# Implementation and Evaluation of Cultural Safety Initiatives in Australian Hospital Settings: A Scoping Review

**DOI:** 10.1177/10436596241296818

**Published:** 2024-11-19

**Authors:** Belinda Clough, Jennifer Fraser, Tara Flemington, Tamara Power

**Affiliations:** 1 The University of Sydney, Camperdown, New South Wales, Australia

**Keywords:** Cultural Safety, Aboriginal and Torres Strait Islander health, First Nations health, Australia, hospitals, literature review

## Abstract

**Introduction:**

The aim of this scoping review was to synthesize and summarize existing evidence for implementing and evaluating Cultural Safety initiatives in Australian hospitals for Aboriginal and Torres Strait Islander peoples. The rationale for this work was to establish guidelines for best practice in providing culturally safe care across hospital and health services.

**Methodology:**

A systematic search guided by PRISMA Extension for Scoping Reviews was conducted across five databases: CINAHL, Scopus, MEDLINE, Embase, and Informit. Reference lists and citations of eligible studies were also examined. Studies were eligible for inclusion if they were in English and discussed the implementation and/or evaluation of Cultural Safety initiatives in Australian hospitals. Evaluations assessing both health care staff and patient perspectives were included. Studies were excluded if they presented future implementation protocols or initiatives among health care students. No date limiters were applied due to the small body of available literature.

**Results:**

Nine studies met the criteria and were included in this review. Five themes emerged: (a) process of implementation; (b) process of evaluation; (c) change in health professional’s behavior; (d) change in patient behavior; and (e) future recommendations.

**Discussion and Conclusions:**

Validated implementation frameworks and evaluation tools are needed for integrating Cultural Safety initiatives into Australian hospital settings. This review has highlighted that despite the availability of evidence-based tools, their use was lacking. This scoping review also highlighted that evaluation of Cultural Safety initiatives is primarily focused on the attitudes and knowledge of health professionals, rather than patient experience. As Cultural Safety is determined by recipients of care, future evaluation of initiatives should prioritize patient perspectives.

The strengths of Aboriginal and Torres Strait Islander peoples, embedded in culture and kinship, endure today despite the profound impact of colonization (Bourke et al., 2018). However, intergenerational trauma resulting from colonization continues to have significant influence on the health and social and emotional wellbeing of Aboriginal and Torres Strait Islander Australians (Smallwood et al., 2021). The impacts are perpetuated by ongoing racism and a lack of Cultural Safety in government systems and services, particularly hospitals (Durey et al., 2012; Parter et al., 2021). Consequently, the actions and responses of health care professionals who engage with Aboriginal and Torres Strait Islander people are critical to ensuring Cultural Safety in hospital settings. This requires attention to clinician knowledge and attitudes in relation to Aboriginal and Torres Strait Islander Australians as well as adoption of strategies that are known to deliver high-quality, culturally safe care. There is an existing strong body of evidence surrounding the development of potentially effective strategies, protocols, and models of care promoting Cultural Safety, such as the Daalbirrwirr Gamambigu (Safe Children) model (Flemington et al., 2022).

However, this body of evidence must be supported by knowledge about the implementation, evaluation, and sustainability of these models of care (Elvidge et al., 2020). Valid and sensitive evaluation tools used within robust implementation frameworks are needed. With this in mind, the objective of this scoping review was to provide insights from the available literature for the implementation and evaluation of Cultural Safety initiatives in Australian hospital settings. The research question was “How have initiatives aimed at improving Cultural Safety for Aboriginal and Torres Strait Islander people been implemented and evaluated in hospitals in Australia?”

## Method

To answer the research question, a scoping review was undertaken guided by the PRISMA Extension for Scoping Reviews (PRISMA-ScR) (Tricco et al., 2018). A scoping review allowed for an adequately comprehensive overview of the recent literature to appraise implementation and evaluation strategies, as well as identify any significant gaps in the knowledge base (Tricco et al., 2018). The search strategy was formulated with librarian input. The protocol was not registered.

### Eligibility Criteria

Studies were eligible for inclusion if they were in English and discussed the implementation and/or evaluation of Cultural Safety initiatives in Australian hospitals. Evaluations assessing both health care staff and patient perspectives were included. Studies were excluded if they presented future implementation protocols, as these have not yet been evaluated, or initiatives among health care students, as implementation of these differed significantly from hospital settings. No date limiters were applied due to the small body of available literature.

### Information Sources

A systematic search was conducted across five databases in June 2023: Cumulated Index to Nursing and Allied Health Literature (CINAHL), Scopus, MEDLINE, Embase, and Informit. Reference lists and citations of eligible studies were also examined.

### Search

The initial search was undertaken by the first author (B.C.). An example of the full electronic search strategy is provided in Table 1. The referencing program Endnote was used to save search results and remove duplicates (The Endnote Team, 2013).

**Table 1.** Search Strategy.

Databases	Inclusion criteria	Exclusion criteria
CINAHL, MEDLINE, Scopus, Embase, Informit	All years English language Australia Hospital setting/hospital workers	Future study protocols (not yet evaluated) Health care students
Search string (conducted on June 6, 2023)		
Indig* OR Aborig* OR First Nation* OR Torres Strait Island* OR First Australian* OR First People* (if subject headings available, also used “Indigenous Peoples+” OR “Aboriginal Australians” OR “First Nations of Australia”) AND cultural* safe* OR cultural* competen* OR cultural* appropriate* OR cultural* capab* OR cultural* sensitiv* OR cultural* aware* OR cultural* understanding OR cultural* respect* OR cultural* responsive OR cultural* humility OR antiracis* OR anti-racis* OR nurse?patient relation* OR physician?patient relation* OR quality improve* (if subject headings available, also used “Cultural Safety” OR “Transcultural Care” OR “Cultural Competence” OR “Antiracism” OR “Nurse–Patient Relations” OR “Physician–Patient Relations” OR “Quality Improvement+” OR “Evaluation and Quality Improvement Program” OR “Attitude of Health Personnel+”) AND Australia OR New South Wales OR Victoria OR Tasmania OR Queensland OR South Australia OR Western Australia OR Australian Capital Territory OR Northern Territory (if subject headings available, also used “Australia+”) AND Hospital (if subject headings available, also used “Hospitals+”)		

### Selection of Sources of Evidence

The titles and abstracts of search results were screened for relevance, followed by reading the full text of promising studies. Screening was undertaken by the first author (B.C.), under the guidance of her supervisory team. Any studies that prompted uncertainty regarding their inclusion in the review were read and discussed by all team members.

### Critical Appraisal of Evidence

Studies were subject to quality appraisal utilizing the appropriate checklist for each study type, as depicted in Tables 2–4. Quality varied across the studies, although the majority were deemed to be of moderate or good quality. However, all results were included in the review regardless of quality due to the insights they could offer regarding the research question.

**Table 2.** AXIS Tool for Cross-Sectional (Quantitative) Studies.

Question	Mooney et al. (2005)	Chapman et al. (2014)
Introduction		
1. Were the aims/objectives of the study clear?	Yes	Yes
Methods		
2. Was the study design appropriate for the stated aims?	Yes	Yes
3. Was the sample size justified?	Yes	No
4. Was the target/reference population clearly defined? (Is it clear what the research was about?)	Yes	Yes
5. Was the sample frame taken from an appropriate population base so that it closely represented the target/reference population under investigation?	Yes	Yes
6. Was the selection process likely to select subjects/participants that were representative of the target/reference population under investigation?	Yes	No
7. Were measures undertaken to address and categorize nonresponders?	Yes	Yes
8. Were the risk factor and outcome variables measured appropriate to the aims of the study?	Yes	Yes
9. Were the risk factor and outcome variables measured correctly using instruments/measurements that had been trialed, piloted, or published previously?	Yes	Yes
10. Is it clear what was used to determine statistical significance and/or precision estimates (e.g., *p*-values, confidence intervals)?	Yes	Yes
11. Were the methods (including statistical methods) sufficiently described to enable them to be repeated?	Yes	Yes
Results		
12. Were the basic data adequately described?	Yes	Yes
13. Does the response rate raise concerns about nonresponse bias?	No	No
14. If appropriate, was information about nonresponders described?	Yes	Yes
15. Were the results internally consistent?	Yes	Yes
16. Were the results presented for all the analyses described in the methods?	Yes	Yes
Discussion		
17. Were the authors’ discussions and conclusions justified by the results?	Yes	Yes
18. Were the limitations of the study discussed?	Yes	Yes
Other		
19. Were there any funding sources or conflicts of interest that may affect the authors’ interpretation of the results?	Unknown	Unknown
20. Was ethical approval or consent of participants attained?	Unknown	Yes
Quality judgment	Moderate	Moderate

**Table 3.** CASP Tool for Qualitative Research Appraisal.

Question	Victorian Department of Health (2010)	Westwood and Westwood (2010)	Kerrigan et al. (2022)
1. Was there a clear statement of the aims of the research?	Yes	Yes	Yes
2. Is a qualitative methodology appropriate?	Yes	Yes	Yes
3. Was the research design appropriate to address the aims of the research?	No	Yes	Yes
4. Was the recruitment strategy appropriate to the aims of the research?	Yes	Yes	Yes
5. Was the data collected in a way that addressed the research issue?	No	Yes	Yes
6. Has the relationship between researcher and participants been adequately considered?	No	No	Yes
7. Have ethical issues been taken into consideration?	No	No	Yes
8. Was the data analysis sufficiently rigorous?	No	Yes	Yes
9. Is there a clear statement of findings?	No	Yes	Yes
10. How valuable is the research?	Low	Moderate	High
Quality judgment	Poor	Moderate	Good

**Table 4.** MMAT Tool for Quality Appraisal of Mixed Methods Papers.

Category	Criteria	Vass (2015)	Gadsden et al. (2019)	Kerrigan et al. (2020)	Rissel et al. (2022)
Screening questions	1. Are there clear research questions?	Yes	Yes	Yes	Yes
	2. Do the collected data allow to address the research questions?	Yes	Yes	Yes	Yes
1. Qualitative	1.1. Is the qualitative approach appropriate to answer the research question?	Yes	Yes	Yes	Yes
	1.2. Are the qualitative data collection methods adequate to address the research question?	Yes	Yes	Yes	Yes
	1.3. Are the findings adequately derived from the data?	No	Yes	Yes	Yes
	1.4. Is the interpretation of results sufficiently substantiated by data?	No	Yes	Yes	Yes
	1.5. Is there coherence between qualitative data sources, collection, analysis, and interpretation?	No	Yes	Yes	Yes
4. Quantitative descriptive	4.1. Is the sampling strategy relevant to address the research question?	Yes	Yes	Yes	Yes
	4.2. Is the sample representative of the target population?	Yes	Yes	Yes	Yes
	4.3. Are the measurements appropriate?	Unknown	Unknown	Yes	Yes
	4.4. Is the risk of nonresponse bias low?	Unknown	Yes	Yes	Yes
	4.5. Is the statistical analysis appropriate to answer the research question?	Unknown	Yes	Yes	Yes
5. Mixed methods	5.1. Is there an adequate rationale for using a mixed methods design to address the research question?	Yes	Yes	Yes	Yes
	5.2. Are the different components of the study effectively integrated to answer the research question?	Yes	Yes	Yes	Yes
	5.3. Are the outputs of the integration of qualitative and quantitative components adequately interpreted?	No	Yes	Yes	Yes
	5.4. Are divergences and inconsistencies between quantitative and qualitative results adequately addressed?	No	Yes	Yes	Yes
	5.5. Do the different components of the study adhere to the quality criteria of each tradition of the methods involved?	No	Yes	Yes	Yes
	Quality judgment	Poor	Good	Good	Moderate

## Results

**Figure 1.**
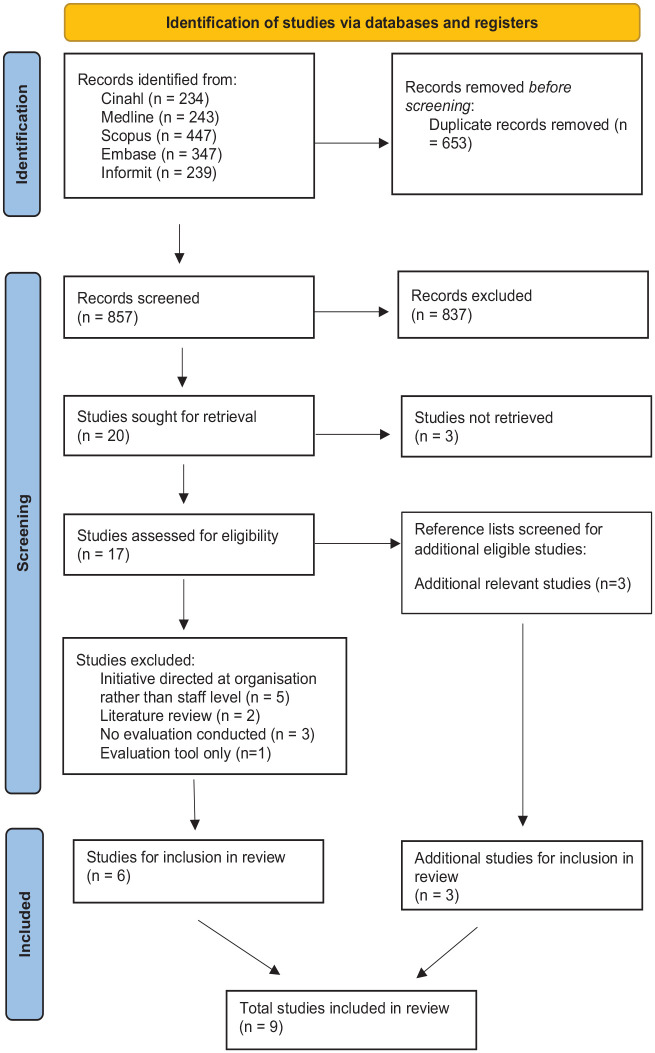
PRISMA Flowchart Depicting Search Results.

The search strategy resulted in 857 studies following the deduplication process, which were then screened according to the process in Figure 1, yielding 9 eligible studies. Excluded studies did not meet the eligibility criteria for this review. A summary of these studies is documented in Table 5. Full text of eligible studies were analyzed thematically drawing on Braun et al.’s (2019) six-step method. This analysis resulted in five themes: the process of implementation, the process of evaluation, change in health professional’s behavior, change in patient experience, and future recommendations.

**Table 5.** Summary of Scoping Review Findings.

Author(s)/year	Cultural Safety initiative	Setting/population	Methodology	Findings
Mooney et al. (2005)	Aboriginal Cultural Awareness Training (CAT), in the form of an in-person, half-day workshop.	84 non-Indigenous health professionals from South Western Sydney Area Health Service (SWSAHS).	• Evaluation via a self-administered questionnaire for participants, developed through consultation and pilot testing. • Questionnaire incorporated quantitative Likert scale questions relating to perceptions, familiarity and friendships, attitudes, and knowledge in regard to Aboriginal patients. • Used “Control” and “Intervention” groups.	• Few measures changed significantly in the intervention group—hence, CAT did not appear to have a major influence on perceptions of beliefs of health staff toward Aboriginal people. • Minor impact on familiarity or friendships with Aboriginal people, as well as increased understanding of complexity of Aboriginal health problems.
Westwood and Westwood (2010)	Aboriginal Cultural Awareness Training (CAT), in the form of an in-person, half-day workshop.	46 senior staff and stakeholders within South Western Sydney Area Health Service (SWSAHS).	• Qualitative evaluation through a review of NSW Health policies and previous evaluations of cultural awareness training initiatives, as well as discussions with relevant staff and stakeholders pertaining to the SWSAHS CAT program.	• No formal published policies on Aboriginal CAT by the NSW Department of Health were identified. • Low attendance rates were reported across CAT programs in SWSAHS. • Evaluations indicated that the content rated well; however, in one evaluation only one-third of participants rated the meeting of the stated objectives as “very good.” • Overall, there was an absence of evaluation or evidence as to whether CAT programs had resulted in more culturally safe care.
Victorian Department of Health (2010)	Cultural Safety quality improvement projects across multiple hospital sites, incorporating developing and facilitating cultural awareness training for staff.	Five Emergency Departments (EDs) in Victoria, and associated staff. Three of these sites implemented or evaluated cultural awareness training.	• Pretraining and posttraining evaluation—no further details provided.	• Increased awareness of Aboriginal and Torres Strait Islander health issues, and improved staff attitudes and knowledge about Aboriginal and Torres Strait Islander culture.
Chapman et al. (2014)	Cultural awareness training conducted by a registered training organization (Kangan Institute). Format was three 2-hr, in-person workshops over 6 weeks, with accompanying learner guide and reflective activities.	44 nursing clerical and allied health ED staff attended and completed surveys.	• Quantitative evaluation via pretest and posttest questionnaire, with participants serving as their own control. • Likert scale questionnaire adapted from Mooney et al. (2005) (listed above)	• Improvement in several responses in regard to perceptions of Aboriginal patients, minor impact on familiarity. • Number of neutral responses decreased, indicating a decrease in ambivalence and a greater willingness to make a definitive choice.
Vass (2015)	The Indigenous Health and Cultural Competency Project, which developed and delivered educational resources regarding First Nations health and culture for emergency medicine doctors. It took the form of a 10-module e-learning series, which included a focus on the practitioner’s own culture.	Emergency doctors who are members of the Australian College of Emergency Medicine.	• Voluntary surveys at the end of each module, incorporating quantitative Likert scales and qualitative responses.	• A low module completion rate. • Not reported how many surveys were completed; however, from the data obtained, modules significantly improved self-assessed knowledge and skills in Aboriginal and Torres Strait Islander health of both trainees and specialists.
Gadsden et al. (2019)	Aboriginal Identification in Hospitals Quality Improvement Program (AIHQIP)—various projects across EDs in NSW. Tailored strategies to each site were developed and implemented with the aim of encouraging Aboriginal patients to identify as Aboriginal in the ED, improving the Aboriginal cultural competence of staff, improving collaboration between the ED and Aboriginal community-controlled organizations, and reducing incomplete ED visits among Aboriginal patients.	Eight public hospital EDs in NSW (mix of rural and metropolitan). The data analysis included Aboriginal people who attended one of the eight participating EDs between January 1, 2010 and March 31, 2015.	• Quantitative indicators—the proportion of Aboriginal patients correctly identified as Aboriginal in ED information systems, and rates of incomplete ED visits. • Qualitative data used for evaluation were interviews with program stakeholders (namely hospital staff) and document review.	• In all EDs combined, the AIHQIP was not associated with a change in the trend of accurate recording of Aboriginality, or a decrease in incomplete ED visits. • Organizational change was achieved throughout the study period, including implementing mandatory cultural training. Staff interviewed identified several factors that they felt enabled program implementation, such as supportive hospital executive staff and a high level of engagement of local Aboriginal community-controlled health services and ALOs in project design and implementation.
Kerrigan et al. (2020)	Aboriginal Cultural Awareness Program (ACAP) delivered by Top End Health Service in the Northern Territory, a 1-day, face-to-face workshop for staff.	596 staff of Top End Health Service, who attended a workshop between March and October 2018, and completed an evaluation survey.	• Voluntary surveys at the end of each workshop, incorporating quantitative Likert scale rankings and free-text qualitative responses. • Thematic analysis of free-text responses was undertaken. • A validated training evaluation model was used as an explanatory framework to assess and interpret the survey results in the discussion, which evaluates training across four levels: (a) learner reactions; (b) learning; (c) on the job behavior change; and (d) observable organizational results.	• Quantitative findings—a mean score of 34/35 was given to the workshop. • Thematic analysis of qualitative data revealed the following theme from participants—a desire for more education, designed and delivered by local people, which provides an opportunity to consciously explore both Aboriginal and non-Aboriginal cultures (including self-reflection).
Kerrigan et al. (2022)	“Ask the Specialist” podcasts—a cultural education package for health care staff.	14 doctors from four hospitals in the Northern Territory.	• Qualitative data via participant weekly reflections, using feedback prompts. • Feedback interviews were also conducted with each participant after they had listened to all podcasts. • Data were analyzed using narrative analysis.	• Five major areas of learning identified, indicating developing critical consciousness as clinicians—the importance of communicating in a culturally safe manner, creating partnerships with patients, awareness of spiritual practices, countering stereotypes, and addressing racism. • Data indicated that critical reflection, prompted by the podcasts, led to behavior change to reduce the power imbalance between patient and clinician in four major areas—investing time to build patient rapport, changing communication, working with interpreters differently, and improving consent processes. • The podcast format was favored by participants for cultural education.
Rissel et al. (2022)	A 1-day cultural awareness training program in Alice Springs, named the “Introduction to Central Australian Cultures and Context.”	2,081 health care staff across 133 workshops over a 5-year period.	• Voluntary surveys at the end of each program, incorporating quantitative Likert scale rankings and free-text qualitative responses. • Qualitative responses analyzed using thematic analysis.	• A very high proportion of respondents reported that the course was relevant to their individual practice or workplace. • From qualitative data, the most useful aspects of the training were education pertaining to historical records and kinship relationships, cultural awareness, presentation and presenters, and interpersonal communication styles. • The least useful aspects of the workshop were that it was not relevant/had already been covered elsewhere, had too much content, some challenging content. • The main learning point was about the history/impact of colonization, followed by a better understanding of local culture and consequent lessons for health care.

### Process of Implementation of Cultural Safety Initiative

Five studies implemented new Cultural Safety initiatives in hospital settings (Chapman et al., 2014; Gadsden et al., 2019; Kerrigan et al., 2022; Vass, 2015; Victorian Department of Health, 2010). Four studies conducted evaluations of preexisting initiatives (Kerrigan et al., 2020; Mooney et al., 2005; Rissel et al., 2022; Westwood & Westwood, 2010), which consequently offered minimal insight into the implementation process.

In examining the nature of the new initiatives, most were implemented across multiple hospital sites (Gadsden et al., 2019; Kerrigan et al., 2022; Vass, 2015; Victorian Department of Health, 2010), with only one study focused on a specific hospital setting (Chapman et al., 2014). A variety of staff were included in the initiatives, including nursing, medical, allied health, management, and clerical staff. It was unclear across all of the studies whether participation in the Cultural Safety training was on a voluntary or a mandatory basis. Most Cultural Safety training was in the form of in-person workshops, presented by Aboriginal or Torres Strait Islander facilitators. Only Chapman et al. (2014) identified that supportive learning materials were also provided and critical reflection was incorporated into the content. Two other initiatives were in the form of e-learning modules or podcasts that could be completed independently, targeted solely at medical officers (Kerrigan et al., 2022; Vass, 2015).

Overall, minimal detail was provided about the process of implementation itself, with the exception of the large-scale Cultural Safety quality improvement project in Emergency Departments (EDs) reported on by Gadsden et al. (2019). Gadsden et al. (2019) outlined in detail a thorough implementation protocol, including a 1.5-day preparation session for working group members workshopping the implementation process, regular site visits from project officers, and regular communication between working groups to share resources and advice.

Working groups were established to oversee implementation in three of the studies, each including at least one Aboriginal or Torres Strait Islander staff member or stakeholder (Gadsden et al., 2019; Kerrigan et al., 2022; Victorian Department of Health, 2010). Only Gadsden et al. (2019) and Kerrigan et al. (2022) identified the underlying theoretical frameworks used to guide their implementation. Vass (2015) alone helpfully identified barriers to implementation of Cultural Safety training, namely the varying levels of baseline cultural knowledge, particularly with international doctors, as well as the challenge of scheduling the training within a busy teaching program for doctors.

### Process of Evaluation of Cultural Safety Initiative

All studies evaluated either a new or preexisting Cultural Safety initiative, although two studies evaluated the same preexisting Cultural Safety initiative in a slightly different way (Mooney et al., 2005; Westwood & Westwood, 2010). Evaluation was predominately undertaken among health professionals, assessing self-reported change in perceptions, attitudes, and behavior regarding First Nations patients, primarily using questionnaires. Use of an overarching evaluation framework was identified only by Kerrigan et al. (2020) and Kerrigan et al. (2022), in which Kirkpatrick’s training evaluation framework was utilized.

Three studies used solely qualitative evaluation (Kerrigan et al., 2022; Victorian Department of Health, 2010; Westwood & Westwood, 2010). Kerrigan et al. (2022) utilized participants’ written reflections and free-text comments, submitted after listening to the Cultural Safety training podcasts. These were then analyzed by both Aboriginal and non-Aboriginal researchers using inductive narrative analysis. The remaining two studies provided minimal detail as to their evaluation techniques; however, the results indicate they were based on informal discussions, email feedback, and pretraining and posttraining surveys from participants.

Two studies utilized quantitative methods only by use of the same questionnaire, developed by Mooney et al. (2005). This questionnaire was developed and pilot-tested in the early 2000s through consultations with key stakeholders and Aboriginal health workers in South Western Sydney Area Health Service (now Local Health District), where the Cultural Safety training was being conducted. The questionnaire contained six statements to measure perceptions, and five statements to measure attitudes toward Aboriginal and Torres Strait Islander people, with a 5-point Likert-type scale to indicate agreement. There were also three “yes/no” questions regarding familiarity or friendship with Aboriginal and Torres Strait Islander people, and two multiple choice questions about Aboriginal and Torres Strait Islander health status. This questionnaire was conducted before and after completing the Cultural Safety training with results comparison. Chapman et al. (2014) utilized this questionnaire in their evaluation of Cultural Safety training, however concluded that the questionnaire may not be sensitive enough in assessing the effectiveness of the initiative.

The remaining studies undertook evaluation using mixed methods (Gadsden et al., 2019; Kerrigan et al., 2020; Rissel et al., 2022; Vass, 2015). Qualitative methods used in these studies included semistructured interviews with participants and free-form survey questions, while quantitative methods were primarily Likert-type scale survey questions.

Other than the tool developed by Mooney et al. (2005), no other studies utilized a validated evaluation tool, but rather used self-developed survey tools tailored to the specific initiative (Gadsden et al., 2019; Kerrigan et al., 2020; Rissel et al., 2022; Vass, 2015). Furthermore, only one study evaluated changes in patient experience following the Cultural Safety initiative. Gadsden et al. (2019) undertook this quantitatively by assessing identification of Aboriginal and Torres Strait Islander patients in the health care sites and rates of incomplete ED visits by Aboriginal and Torres Strait Islander patients. It was however identified by the authors that these indicators were likely not sensitive enough as a measure of Cultural Safety.

### Change in Health Professionals’ Practice

The resulting changes in health professional’s practice were undoubtedly dictated by the effectiveness of both the implementation and evaluation strategies. Three studies indicated very minimal or no change in health professional’s practice following participation in the Cultural Safety initiative (Gadsden et al., 2019; Mooney et al., 2005; Westwood & Westwood, 2010). The remaining studies found overall improved attitudes, awareness, skills, confidence, and self-assessed knowledge in relation to Aboriginal and Torres Strait Islander health and patient care, to varying degrees. Two of the studies also reported feedback indicating that the Cultural Safety training was perceived to be highly relevant and valued by participants (Kerrigan et al., 2020; Rissel et al., 2022).

### Change in Patient Experience

Evaluation of the Cultural Safety initiative in terms of patient experience was not assessed in eight of the nine studies. Gadsden et al. (2019) analyzed changes in the proportion of incomplete ED visits made by Aboriginal and Torres Strait Islander patients at their site and reported very minimal change. The Victorian Department of Health (2010) large-scale Cultural Safety project also identified that positive feedback was received from local Aboriginal and Torres Strait Islander communities regarding some of the changes initiated but no further detail was provided regarding how this feedback was obtained or measured.

### Future Directions

All studies provided a number of recommendations for future implementation and evaluation of Cultural Safety initiatives. Regarding implementation, recommendations were focused on strategies to improve engagement of hospital staff with the initiatives, such as providing staff Cultural Safety training on a shorter, regular (rather than one-off) basis during work hours, or alternatively in an online self-directed format (Kerrigan et al., 2020, 2022; Westwood & Westwood, 2010). Suggestions in relation to future evaluations of Cultural Safety initiatives focused on the ongoing need for rigor in evaluation, ideally utilizing both qualitative and quantitative methods with both hospital staff and patients (Rissel et al., 2022). More specifically, it was recommended that further research be conducted into appropriate wording and sensitivity of survey questions to ensure accurate evaluation.

## Discussion

This scoping review contributes valuable context to current implementation and evaluation strategies of Cultural Safety initiatives in Australian hospitals. First, it is evident from the findings that evaluation remains primarily focused on staff perspectives rather than patient experience of Cultural Safety. Jongen et al. (2018) also identified this theme in a similar review with an international scope. They concluded that the impact of Cultural Safety initiatives for patients remains unclear, as evaluation is focused on changes in health practitioner knowledge and attitudes. This approach to evaluation neglects the important premise underlying Cultural Safety—that it can only be defined by the recipient of care (Australian Human Rights Commission, 2018). This finding highlights the need to prioritize patient perspectives when evaluating the effectiveness of Cultural Safety initiatives.

However, this must be carefully balanced with the reframing of the concept and practice of Cultural Safety in recent years to focus attention on the need for critical reflection of the health practitioner, while still acknowledging that Cultural Safety can only be defined by the recipient of care (Curtis et al., 2019). Critical consciousness and reflection from the health practitioner are essential to ensure awareness of their own cultural biases and assumptions that may impact on care (Curtis et al., 2019). Therefore, evaluation of Cultural Safety initiatives must encapsulate both changes in both the health practitioner, in regard to incorporating critical self-reflection into their care, and Aboriginal and Torres Strait Islander peoples’ perceptions and experiences of the care received. Although most studies to date did not adopt both of these forms of evaluation, this two-pronged approach is outlined in a recent protocol by Ralph et al. (2023) for the implementation of Cultural Safety initiatives in a Northern Territory hospital setting, in which qualitative and quantitative evaluation from both health professionals and patients will be undertaken in accordance with Cultural Safety principles. This presents a promising model for evaluation for other future Cultural Safety initiatives.

This scoping review also highlights the need for utilization of validated tools in evaluation of Cultural Safety initiatives in hospital settings, as the findings revealed their use was lacking in favor of self-developed tools. This theme was also identified in the international scoping review conducted by MacLean et al. (2023) across health, education, and social work disciplines, which revealed a lack of consistency in the objectives and methods used to evaluate Cultural Safety initiatives. To this end, several new validated evaluation tools have emerged in the wider literature for potential use in this context. For example, Brumpton et al. (2023) have published a recent protocol outlining the planned evaluation of a Cultural Safety initiative among both staff and patients in a general practice setting. Attitudinal change among staff will be assessed with validated survey tools from Ryder et al. (2019) and West et al. (2017), while patient evaluation will occur via semistructured interviews. These tools were also endorsed by Ganeshananthan et al. (2022) in their limited rapid review of self-assessment tools for assessing Cultural Safety. However, for instances where patient interviews are not practicable or possible, Elvidge et al. (2020) have also developed a survey tool to assess patient experience of Cultural Safety in hospitals.

The rigor in validation of these tools and alignment with more developed conceptualizations of Cultural Safety makes them a recommended option for future implementation and evaluation of Cultural Safety initiatives. However, consideration should still be given to self-developed evaluation methods specific to the Cultural Safety intervention, as utilized by the majority of studies, as well as the possibility of adapting the aforementioned validated tools to the specific learning outcomes of the initiative. This approach allows for tailoring of the evaluation tools to the specific setting, population, and initiative.

These evaluation tools should sit under a validated theoretical framework to guide implementation and evaluation, as this was also found to be lacking in the scoping review findings. The implementation process of Cultural Safety initiatives occurred in a mostly ad hoc manner without guidance of an evidence-based framework or theoretical principles. An example of one such framework is the Ngaa-bi-nya Aboriginal and Torres Strait Islander program evaluation framework developed by Williams (2018), which is underpinned by Aboriginal and Torres Strait Islander knowledge and research ethics. It aims to provide a practical guide for evaluating Aboriginal and Torres Strait Islander health and social programs. The framework provides prompts to consider throughout the implementation and evaluation process, and again recommends a mix of quantitative and qualitative methods for evaluation (Williams, 2018). Utilization of frameworks such as Ngaa-bi-nya would help ensure an evidence-based and rigorous implementation and evaluation, guided by Aboriginal cultural knowledge.

Finally, attention needs to be given to the implementation of Cultural Safety initiatives in fast-paced contexts where health care practitioners have considerable preexisting demands on their time and energy—a barrier identified in several studies (Kerrigan et al., 2020; Vass, 2015). Implementing these initiatives in an online self-directed format such as the podcasts evaluated by Kerrigan et al. (2022) can go some way in addressing this barrier. It also allows for Aboriginal and Torres Strait Islander voices and stories to be heard by a wider audience. This makes it a valuable option to be considered in future research.

### Strengths and Limitations

This scoping review contributes valuable evidence to this research area, as no other reviews have focused on implementation and evaluation of Cultural Safety initiatives in the Australian hospital context—a setting where Aboriginal and Torres Strait Islander people continue to experience culturally unsafe care (Durey et al., 2012). Nevertheless, it must be acknowledged that the review was limited by the quality of the relatively small number of studies. Utilizing validated quality appraisal checklists, less than half of the 12 studies were evaluated to be of good quality, which should be considered when examining the findings. In addition, only one of the authors (T.P.) is a First Nations person, descendant of the Wiradjuri people. Other authors, including the first author (B.C.) and other authors (J.F. and T.F.) are non-Indigenous, Caucasian Australian citizens. We acknowledge the impact this may have had on the study.

### Recommendations and Conclusion

The current body of evidence, as analyzed by this review, indicates that significant improvement is needed in adopting evidence-based and carefully considered approaches to implementing and evaluating Cultural Safety initiatives in hospital settings. Specifically, implementation should be underpinned by a validated theoretical framework and consider and address potential practical barriers in engaging health practitioners. It is recommended that evaluation also utilizes validated evaluation tools and incorporates quantitative and qualitative methods to assess changes in both staff and patient perspectives and experiences. This will encapsulate the important notion that Cultural Safety is determined by recipients of care. These valuable insights will guide the implementation and evaluation of future Cultural Safety initiatives in Australian hospitals, to augment efforts to promote Cultural Safety for Aboriginal and Torres Strait Islander families accessing health care services in Australia.
